# Influencing Factors for the Acceptability of Accessing HIV Pre‐exposure Prophylaxis via Community Pharmacies in Wales

**DOI:** 10.1111/hex.70247

**Published:** 2025-04-03

**Authors:** David Gillespie, Adam D. N. Williams, Richard Ma, Zoe Couzens, Kerenza Hood, Dyfrig A. Hughes, Efi Mantzourani, Eleanor Cochrane, Fiona Wood

**Affiliations:** ^1^ Centre for Trials Research, School of Medicine, College of Biomedical & Life Sciences Cardiff University Cardiff UK; ^2^ Centre for Academic Primary Care, Bristol Medical School University of Bristol Bristol UK; ^3^ Public Health Wales NHS Trust Cardiff UK; ^4^ Centre for Health Economics and Medicines Evaluation Bangor University Bangor UK; ^5^ School of Pharmacy, College of Biomedical & Life Sciences Cardiff University Cardiff UK; ^6^ Health Education and Improvement Wales Cardiff UK; ^7^ Division of Population Medicine and PRIME Centre Wales, School of Medicine, College of Biomedical & Life Sciences Cardiff University Cardiff UK

**Keywords:** community pharmacy, healthcare access, HIV, pre‐exposure prophylaxis, primary care, qualitative research

## Abstract

**Introduction:**

HIV prevention methods, such as pre‐exposure prophylaxis (PrEP), have been a significant contributing factor to a global decline in HIV transmission. PrEP has been available through the NHS in Wales since 2017. However, access is exclusively via sexual health clinics, and those accessing PrEP do not reflect those being diagnosed with HIV. Widening access beyond sexual health clinics may be one approach to encourage more equitable uptake, and there is growing interest in offering PrEP services in community pharmacies. We, therefore, aimed to explore the acceptability of PrEP services being delivered through community pharmacies among prospective service users.

**Methods:**

We conducted a qualitative interview study of people living in Wales who either (i) currently access, (ii) previously accessed or are (iii) considering accessing PrEP via a sexual health clinic. Participants were recruited via community networks, and interviews were conducted virtually. Our topic guide was informed by Levesque's conceptual framework of access to healthcare, and we used reflexive thematic analysis.

**Results:**

We interviewed 24 participants and included data from 20 in the analysis. Four themes were generated: experiences of accessing PrEP via sexual health clinics, the prospect of PrEP access via community pharmacies, other community settings in which PrEP may be accessed and concerns around integrated healthcare and healthcare data.

**Conclusion:**

PrEP access via community pharmacy is likely to be an acceptable option for people. There are uncertainties surrounding what a PrEP service would look like in a community pharmacy setting, and this would need clarifying to prospective users to increase the salience of access.

**Patient and Public Contribution:**

A team‐based approach was taken for developing the topic guide and agreeing on the codes for this study. This included people with lived experience of accessing PrEP in Wales.

## 1 Introduction

While HIV remains a global health concern [1], the increase in the availability of pharmacological HIV prevention methods over the past decade, such as pre‐exposure prophylaxis (PrEP), has been a significant contributing factor to a global decline in HIV transmission [2].

PrEP involves the use of antiretroviral medication to prevent HIV acquisition. It is highly effective in preventing HIV acquisition in several populations [3, 4, 5, 6]. Oral PrEP (Tenofovir disoproxil fumarate) has been available through the NHS in Wales since 2017 [7]. Since then, it has been prescribed to > 3000 individuals (correct as of the end of 2022) exclusively through sexual health clinics. Most individuals being prescribed PrEP in Wales are male (99%), of white ethnicity (69%) and identified as men who have sex with men (81%).

There is a mismatch between the demographics of people being diagnosed with HIV and those accessing PrEP [8]. Difficulties with access and stigma associated with sexual health clinics are often cited as reasons for not engaging in sexual healthcare [9, 10, 11]. There is growing interest in alternative options, such as remote options and primary care services like general practice and community pharmacies [12].

The HIV Action Plan for Wales aims to implement a shared care model for PrEP access between primary care and sexual health services [13]. Community pharmacies contribute to the health and well‐being of the population by delivering clinical services. NHS Contracted Services delivered through community pharmacies in Wales include (among others) the common ailments scheme, emergency contraception services, seasonal flu vaccination, urinary tract infection service and the sore throat test and treat service [14]. Some of these services have shown sharp increases over the years since their introduction, and evidence suggests that these services are acceptable to users [15, 16]. While there are several examples of sexual healthcare delivery via community pharmacies to complement those delivered by sexual health clinics, we do not know if PrEP provision is feasible and acceptable to individuals and why they might choose community pharmacies over other services. This study aims to address these questions.

## 2 Materials and Methods

### 2.1 Study Design and Theoretical Framework

We used a phenomenological approach for this semi‐structured interview study of people living in Wales who currently, previously or may in the future access PrEP.

We drew on Levesque's conceptual framework of access to healthcare (2013) and defined access as ‘the opportunity to reach and obtain appropriate healthcare services in situations of perceived need for care’ [17]. Within this framework, there are five dimensions of accessibility (approachability, acceptability, availability and accommodation, affordability, and appropriateness) and five dimensions of abilities that potential service users require (ability to perceive, seek, reach, pay and engage).

The study was reviewed and approved by the Cardiff University School of Medicine Research Ethics Committee (Reference Number: 23/91).

### 2.2 Participant Selection

Drawing on the 2023 PrEP need framework, which identifies eight different forms of need associated with PrEP [18], we interviewed individuals who: (i) currently access PrEP through a sexual health clinic in Wales (Group 1); (ii) have previously accessed PrEP through a sexual health clinic in Wales (Group 2); (iii) are resident in Wales, aware that they might benefit from PrEP (through awareness that it is an HIV prevention option and their own self‐assessment of risk and/or need), but had not accessed it through a sexual health clinic in Wales (Group 3). Inclusion criteria were those who were over 18 years old, belonged to one of the three target populations above, were able to communicate fluently in English (though English did not need to be their first language) and gave consent.

### 2.3 Setting and Data Collection

Participants were recruited from the community via social media advertising and other social networks (e.g., Fast Track Cymru [19], THT Cymru [20], PrEPster [21], local councils and networks of PrEP users from associated research). We advertised the study in NHS settings (e.g., sexual health clinics, GP practices and community pharmacies) using posters, but we did not recruit directly from these services. To acknowledge their time, participants were offered a £20 digital gift voucher.

The lead researcher (D.G.) sent an information sheet and consent form to prospective participants. With consent, online interviews were conducted by either D.G. or A.W. using a topic guide that covered PrEP access history and acceptability of PrEP services delivered via community pharmacy and other potential community settings. The topic guide can be viewed in Appendix 1.

We interviewed 24 individuals but planned for 45 (15 per target population group), based on the information power model proposed by Malterud (2016) [22]. We used maximum variation sampling to incorporate views from individuals of different genders, ethnic groups, ages and individuals from different parts of Wales.

### 2.4 Data Analysis

All transcripts were checked against the recording for accuracy and anonymised. We conducted a reflexive thematic analysis to analyse our interview data [23]. Following familiarisation with the data, codes were developed by inspecting transcripts line by line, with an initial coding framework developed. Double coding was supported by F.W. and R.M. for the first five interviews to agree on the initial coding framework, accounting for alternative perspectives. Subsequently, a further five interviews were double coded to assess coding consistency. Themes were developed using the ‘One Sheet of Paper’ or ‘OSOP’ technique and were reviewed, refined and subsequently named.

During our recruitment process, we suspected that we may be prone to so‐called imposter participants (i.e., participants pretending to meet eligibility criteria) [24]. We (D.G., A.W. and F.W.) therefore reviewed the timing of expressions of interest, consent forms, audio from interviews and interview transcripts. If concerns were present across all four of these elements (e.g., the expression of interest was sent near in time to others, consent forms were completed almost identically, cameras would not or could not be used for the interview and answers were vague), participants were considered imposters and excluded from the analysis. Where there were concerns around some but not all elements, these were discussed, and a consensus was reached about how their data would be handled.

### 2.5 Research Team and Reflexivity

All four researchers directly involved in data collection and analysis are experienced in research around PrEP. The team was supported by others with expertise in community pharmacy practice, public health policy and lived experience of accessing PrEP in Wales. Our core data collection and analysis team consisted of males and females, straight and gay, clinicians and non‐clinicians, and those with an age range of 27–55. Our team‐based approach to developing the topic guide and codes encouraged alternative views and perspectives. This study was conducted in response to both stakeholder engagement work, which highlighted the role that community pharmacies could play in expanding access to PrEP, and an action within the Wales HIV Action Plan, which indicated that access to PrEP would be expanded beyond sexual health clinics in Wales.

## 3 Results

### 3.1 Participants

We interviewed 24 participants between February and June 2024. Four participants were deemed imposters by the research team and were discarded from our analysis. The remaining five participants were considered potential imposters but retained following agreement that the guarded nature of their responses could be explained by the nature of the research. Data from these five interviewees were given less weight in our analysis. We therefore included 20 participants in our analysis. Our sample included people with varying levels of experience of accessing PrEP as well as diversity in terms of age, gender identity, ethnic group and area of residence in Wales (Table 1).

**Table 1:** Demographic characteristics of participants included in the analysis (*N* = 20).

Question	Response	Frequency	%
PrEP experience	Currently accessing PrEP in Wales	9	45
	Previously accessed PrEP in Wales	7	35
	Aware that they may benefit from PrEP but not yet accessed in Wales	4	20
Region of Wales	Cardiff	14	70
	Outside Cardiff*	6	30
Age group	18–24 years	0	0
	25–30 years	10	50
	31–40 years	7	35
	41+ years	2	10
	Missing	1	5
Gender identity	Man (including trans man)	14	70
	Woman (including trans woman)	4	20
	Missing	2	10
Ethnicity	White	9	45
	Non‐white**	9	45
	Missing	2	10
Sexual orientation	Heterosexual	10	50
	Gay or bisexual	9	45
	Missing	1	5

* Including Bridgend, Conwy, Newport and Rhondda Cynon Taf.

** Including Asian British, Black, Black British, Black American and Caribbean.

**Figure 1:**
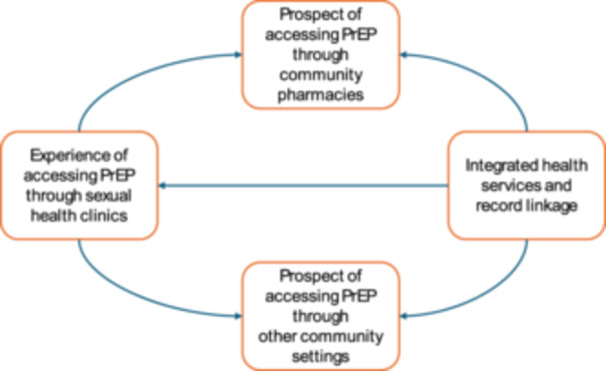
Themes generated around the acceptability of accessing PrEP via community pharmacies in Wales.

We generated four themes, the most pertinent capturing the experiences of accessing PrEP via sexual health clinics, the perceived influences on and acceptability around accessing PrEP via a community pharmacy, as well as other community‐based settings. However, an additional theme was identified covering the integration of health services and associated data. Figure 1 illustrates the generated themes and their conceptual links. Thematic matrices can be viewed in Appendix 2.

#### 3.1.1 Experiences Accessing PrEP Through Sexual Health Clinics

For participants who recognised that HIV transmission in Wales is primarily through sexual contact, the sexual health clinic was deemed an appropriate setting in which PrEP would be available. However, difficulties and inconsistencies in access across health boards were noted. Some participants felt more comfortable than others accessing PrEP from sexual health services. Our first theme is therefore divided into two sub‐themes: the first covering the process of accessing the sexual health clinic itself and the second covering anticipated and enacted stigma and discrimination around PrEP access via sexual health clinics.

#### 3.1.2 Process of Getting PrEP Through a Sexual Health Clinic

Participants described a mixture of routes to accessing PrEP, including opportunistic signposting from the sexual health clinic, friends/family who knew about PrEP and their own research. One participant described signposting from their GP, as an indicator of the willingness of these settings to refer people to PrEP services should the request arise.

‘I'm a sex worker. So, I need to protect myself, because I do have a lot of clients with several requests. And in my line of work, I have a very high risk of contracting the virus (HIV). So, I have to take tests to protect myself, and one of these tests was getting PrEP. I have unprotected sex with my clients, and this was readily available to me when I do have sex, through my local community Sexual Health Clinic.’

PID 3, Group 2, Outside Cardiff, Aged 25–30, non‐White, Man, Straight

Difficulties in accessing PrEP were noted. These included the operating hours of clinics being challenging for those with 9–5 jobs and pathways being cumbersome. The latter was exacerbated by the knowledge that pathways differed across health boards.

‘…the hardest thing for me was being able to attend the clinic, because I work Monday to Friday basically nine until five and I think the clinic is open nine until three and it's not open five days a week, or it wasn't at the time. The only way for me to actually be able to attend in‐person was to take time off work.’

PID 1, Group 2, Cardiff, Aged 25–30, White, Man, Gay or bisexual

‘I had to go to one hospital to have the check‐up and to have the request for it, and all of those things…they gave me a prescription, but then I had to go to another hospital (to) their pharmacy to collect it…they were giving three months' supply, so…every three months I have to go through the same process.’

PID 5, Group 1, outside Cardiff, Aged 41–50, White, Man, Gay or bisexual

However, some participants described the process of accessing PrEP services as positive.

‘I think trying to get (access to) the service…from finding (out) about it and researching and booking the call and being able to find appointments, the process was not stressful so that's kind of a positive.’

PID 14, Group 1, outside Cardiff, Aged 31–40, non‐White, Man, Gay or bisexual

#### 3.1.3 Stigma and Discrimination Around PrEP Access via Sexual Health Clinics

There was a mixture of views regarding the stigma around accessing PrEP via a sexual health clinic. This seemed to focus on the level of comfort participants had with sharing details of their sexual practices and sexual orientation with sexual health clinic staff, with participants describing initial encounters with staff as particularly discomforting.

‘I don't like to go there and get lots of questioning, it's one of the barriers, that's kind of, hindering me.’

PID 4, Group 2, Cardiff, Aged 25–30, non‐White, Woman, Straight

‘I didn't enjoy the whole communication in seeking out (information) because they made me really focus on so many personal things such as my sexuality…I think the whole process of the referral from a sexual health clinic shouldn't be necessary because they (clinic staff) see it as an opportunity to ask a whole lot of questions that I don't think is necessary.’

PID 2, Group 2, Cardiff, Aged 25–30, non‐White, Man, Straight

While this was not the case for all participants, those who experienced less discomfort sharing this information with clinic staff still reflected that this may influence access to PrEP for others.

‘I know that there is still a stigma attached to sexual health clinics. I don't know whether having to get PrEP through a sexual health clinic would put people off. Not wanting to just…(share) their sexual orientation, or the fact that they are engaging in sexual experiences that might require PrEP.’

PID 19, Group 1, Cardiff, Aged 25–30, White, Man, Gay or bisexual

Despite any discomfort when discussing sex or being directly asked about their sex lives, there was an acceptance that HIV was primarily acquired through sexual contact and that, therefore, sexual health clinics were able to support people to access PrEP by employing staff who were trained to provide relevant testing and medication.

‘Well, it's (having access to PrEP in a sexual health clinic) positive because…I would assume the most common way for the virus to be transmitted is through sexual means. So, it absolutely makes sense that the PrEP medication would be available at the Sexual Health Clinic.’

PID 3, Group 2, outside Cardiff, Aged 25–30, non‐White, Man, Straight

#### 3.1.4 Prospect of Accessing PrEP Through Community Pharmacies

Turning attention to the primary aim of this study, our analysis developed three sub‐themes covering recognition of community pharmacy as a healthcare setting, experiences of service delivery through community pharmacy and the acceptability of a PrEP service delivered through community pharmacy. To date, PrEP in Wales has been delivered through sexual health services, so discussions about community pharmacy PrEP services were hypothetical.

#### 3.1.5 Awareness of Community Pharmacies as a Healthcare Setting

Participants had a clear understanding of the role of community pharmacies. Primarily, participants identified the dispensing of medication as their primary function, though some also identified other clinical services provided, such as influenza vaccinations. Some participants were aware of a ‘shift’ in clinical services from other primary care settings.

‘I've noticed recently that services that I would normally have gone to my GP for have been deferred to my pharmacy…I would think it's (community pharmacy) where I go to get my prescription, it's where I could go for some advice if I don't need to go to A&E and I don't feel like I desperately need to see a doctor, but I probably wouldn't go there for much else.’

PID 20, Group 3, Cardiff, Aged 31–40, White, Man, Gay or bisexual

A notable distinction was made between community pharmacies and other NHS‐based healthcare settings with regard to it being a ‘business’. This business model of healthcare service implied that it could be regarded as a retail setting. There could also be implications for the trust placed in certain pharmacies owing to their ‘business‐like’ nature (e.g., the perception of there being a conflict between giving health advice and selling over‐the‐counter treatments and personal care products).

‘I do have a distinction between community pharmacy and the NHS…The NHS is a public service. Whereas the community pharmacy is a private sector…If I wanted a quality service from a community pharmacy, I would go to (major high‐street community pharmacy). I trust (major high‐street community pharmacy) more than the community pharmacy I use… …Community pharmacy to me…it's a shop that sells things.’

PID 6, Group 1, Cardiff, Aged 41+, White, Man, Gay or bisexual

#### 3.1.6 Experience of Service Delivery Through Community Pharmacies

There was a strong sense of ‘easy access’ in terms of location, opening hours and days of the week on which community pharmacies are open. The ‘walk‐in’ nature was appealing when compared to other settings, and it meant that the service fitted in with peoples' lives rather than vice versa.

‘The one closest to me is open on a Saturday, so if I can't get there Monday to Friday, I can at the weekends.’

PID 1, Group 2, Cardiff, Aged 25–30, White, Man, Gay or bisexual

However, concerns were raised regarding medication stock supplies. Participants drew on experiences where they were told the medication was not in stock. With regularity, this can create issues of trust in a service's ability to deliver. For PrEP, stock issues could mean that people had an insufficient supply to cover periods of risk exposure, thus reducing PrEP's effectiveness.

‘…then you come and it's (medicine) not available. This was I think last month. I came to get a particular medication, and they didn't have it. They are usually out of medication really quick.’

PID 14, Group 1, outside Cardiff, Aged 31–40, non‐White, Man, Gay or bisexual

Despite this, there was a sense that the familiarity of staff in a local pharmacy, in addition to the varied nature of attendance (which could include visiting a pharmacy to make retail purchases), made it a relaxed healthcare setting relative to others. This familiarity created a sense of implicit trust and increased the salience of using community pharmacies.

‘I think a pharmacy has less people and…being somewhere you go for a different purpose, you might feel much more comfortable in that kind of place…I think the familiarity is there and it makes it easier to feel much more comfortable.’

PID 11, Group 1, outside Cardiff, Aged 31–40, non‐White, Man, Straight

#### 3.1.7 Delivery of a ‘PrEP Service’ Through Community Pharmacies

When discussions turned to the delivery of a ‘PrEP service’ via community pharmacies, there were mixed views from participants, and this tended to stem from the varied understanding of what a ‘PrEP service’ comprised.

In terms of a PrEP collection service from a community pharmacy, this was viewed as both acceptable and a welcome change compared to travelling to pick up a prescription from a sexual health clinic or, in some cases, travelling between sexual health clinics to receive a complete PrEP service. The key features which made this acceptable were the convenience of accessing a community pharmacy for this purpose and the varied and confidential nature of collecting a prescription from a pharmacy meaning that this could be done with anonymity. These aspects were thought to enable wider access to PrEP.

‘If it (PrEP) were to be delivered from pharmacies…people would be able to access it easily.’

PID 16, Group 3, Cardiff, Aged 25–30, non‐White, Woman, Straight

Participants noted a difference between collecting PrEP medications and getting sexual health and/or PrEP advice from pharmacists, with the latter being more challenging for some.

‘…that's different to going to the pharmacy to pick up a prescription, that's understood, known, dispensed, and you're just picking up a package, because you could be picking up anything then, it doesn't really matter. If I was to go to the pharmacy and try to have a conversation I might be having at the sexual health clinic in the pharmacy, I think that might be challenging.’

PID 5, Group 1, outside Cardiff, Aged 41+, White, Man, Gay or bisexual

Participants highlighted that the drawback of the ‘local’ nature of community pharmacies is that you are in your local community. In this context, sharing information about your sexual activity in such a setting may be unacceptable to some.

‘I wouldn't feel so comfortable having an STI screening in a community pharmacy. Mainly because of the proximity…I'd prefer to go a 30 minutes' drive to get my STI screening than walk into a pharmacy.’

PID 14, Group 1, outside Cardiff, Aged 31–40, non‐White, Man, Gay or bisexual

Some participants raised concerns regarding the willingness of pharmacists to positively engage in PrEP services, drawing on reluctance around emergency contraceptive services. This perceived reluctance was considered to stem from religious beliefs and anticipated PrEP‐related stigma.

‘I worry that there might be some pharmacists that wouldn't do the training because of the stigma associated with HIV…there are some pharmacists that refuse to do the morning after pill because if they're Catholic or Muslim it goes against religion…And I worry that would happen with PrEP as well, because it could be seen as men having sex with men…’

PID 8, Group 2, Cardiff, Aged 31–40, White, Man, Gay or bisexual

There were concerns that certain facilities are not available in community pharmacies to enable a complete PrEP service inclusive of STI testing, other testing (e.g., liver/kidney function) and space for sexual health and adherence counselling. This perceived lack of facilities would lead some to avoid accessing PrEP services in community pharmacy, even components of a service, in favour of a complete service in a single less‐convenient setting.

‘I would want to know how it would work, because the first time I went on PrEP, I had the blood test taken and I don't feel that can be done at a pharmacy…it was useful for me to go to the sexual health clinic because I was able to knock out everything at once. I could see that being an issue at pharmacy, where you couldn't get everything integrated….as well I was able to pick up contraceptives at the (sexual health) clinic too after I'd had all the tests…It'd be like here's your PrEP, here's your condoms, have a great time.’

PID 19, Group 1, Cardiff, Aged 25–30, White, Man, Gay or bisexual

However, among others, provided that the facilities were available, they would be willing to undertake services in their community pharmacy.

‘I can't think I'd have a problem with that (STI testing services) being in a pharmacy setting. It comes back to the same thing if you trust the practitioner who is helping you and you need the service, just do it…It would be a little awkward sometimes but you just get on with it for the sake of your health.’

PID 20, Group 3, Cardiff, Aged 31–40, White, Man, Gay or bisexual

The training of community pharmacy staff in the delivery of a PrEP service was highlighted as an important issue. While it was mentioned that this training should cover knowledge and skills (e.g., about PrEP and how to conduct necessary tests), emphasis was also placed on training staff to deliver a non‐judgemental service sensitive to the needs of its recipients. The ability to create this ‘welcoming environment’ was considered to engender trust in the service, which would make people more willing to access it. Linking back to the first theme around PrEP services in sexual health clinics, this ‘environmental’ aspect was considered a key differentiating feature of the expectation of the type of service a recipient would receive in a community pharmacy compared to a sexual health clinic. Specifically, sexual health clinics have staff who are trained and primed to have non‐judgemental and inclusive discussions about sexual health, whereas community pharmacies are a more general setting and may be less primed.

‘I wouldn't have a problem with having that conversation (about PrEP) with somebody there (a community pharmacy) as long as I had faith that they (were) trustworthy and non‐judgemental and gave me a private space to have that conversation openly.’

PID 20, Group 3, Cardiff, Aged 31–40, White, Man, Gay or bisexual

#### 3.1.8 Prospect of Accessing PrEP Through Other Community Settings

Several other community settings were suggested as places where PrEP services could be delivered. These are split into four types: general practice, other healthcare settings, non‐health community settings and digital or de‐centralised services.

#### 3.1.9 PrEP Delivered via General Practice

In addition to community pharmacies, some participants discussed the prospect of receiving a PrEP service via their general practice. It was surprising that this was not being actively pursued as an option, particularly given its similar nature to community pharmacy in terms of location and recognised availability of important facilities. Participants also proposed the idea of a primary care pathway which integrated general practice (e.g., for consultation and testing) and community pharmacy (for collection).

‘…it feels a bit backwards, the fact you can get the contraceptive pill through GP, whereas you can't get PrEP through GP. Blood test wise you, you can get that easily through the GP surgery…so it (PrEP) could easily be retrieved from the community pharmacy’

PID 8, Group 2, Cardiff, Aged 31–40, White, Man, Gay or bisexual

#### 3.1.10 Other Specialist Health Services

In terms of other specialist health services, participants proposed PrEP delivery via gender identity clinics. The salient feature of a service via this route was the expectation of a service that had staff who were trained in trans‐inclusivity, with the implication being that trans individuals may have concerns about accessing services where this is lacking.

‘…for a lot of trans‐people, talking to a clinician that you know is well‐trained in trans‐identity is possibly the only way that some trans‐people might consider accessing PrEP…other less specialist services, whether it is your GP or your sexual health clinic, or your pharmacy, it does always feel like more of a risk of putting yourself out there in a position where you might have to educate the people that are supposed to be more educated than you about that kind of medical thing that you're seeking support with.’

PID 1, Group 2, Cardiff, Aged 25–30, White, Man, Gay or bisexual

Another proposal was for PrEP to be delivered via mental health services. A useful feature of such a service was the ability to receive counselling and consultation, which could identify potential sexual risks.

‘Sometimes someone that is suffering from a sexual illness can have mental sickness or mental issues that affect his or her thinking……apart from the PrEP services they can give an avenue for counselling, consultation and examination.’

PID 10, Group 1, Cardiff, Aged 31–40, non‐White, Man, Straight

Community‐based organisations, such as hubs/libraries or supermarkets, were additional settings suggested for the delivery of PrEP services. It was felt that these settings would inherently de‐medicalise PrEP, which may be appealing to some prospective users. Key motivations underpinning these suggestions appeared to be improved access (in terms of geography) and precedents set around other medications used to enhance sexual health and well‐being.

‘Supermarkets…I mean there are many precedents, if you think about erectile dysfunction and you can now buy Viagra or the equivalent off the shelf. And, I believe Viagra can have some adverse side effects, particularly those that have blood pressure issues, or those that combine use of Viagra with recreational drugs…But I assume that somehow they've overcome that barrier by selling them on supermarket shelves*…if, PrEP is safe, which I think it is, and if PrEP can result in improvement or safeguard the individual health and general collective public health, why wouldn't you make it as open and accessible to everyone?’

PID 6, Group 1, Cardiff, Aged 41+, White, Man, Gay or bisexual

*NB. In the United Kingdom, Viagra is classified as Pharmacy Medicine and can be bought only from pharmacies (including supermarkets with on‐site pharmacies) and under a pharmacist's supervision.

A final area mentioned during interviews was digital services and the broader notion of decentralised PrEP services. Two specific examples of this were given during interviews: digital services and the concept of a ‘home‐based’ PrEP service, as well as mobile or travelling clinics. Key advantages of these were that they brought services to people and may be useful to individuals in remote areas.

‘You (could) do it (access PrEP) in the convenience of your home and order the drug to your home address…So more like test kits being posted to your home address, conveniently would help. It would ensure that you're always on track about your PrEP medication. It will increase aspects of healthcare and eliminate waste, waiting times…the advantages outweigh the disadvantages.’

PID 2, Group 2, Cardiff, Aged 25–30, non‐White, Man, Straight

‘…banks have closed down and in my small village, a bank van comes out once a week and you know what time it gets there, it stops in the layby, and for two hours it's there, and you could quite easily have a sexual health van.’

PID 6, Group 1, Cardiff, Aged 41+, White, Man, Gay or bisexual

#### 3.1.11 Integrated Health Services and Record Linkage

A potential structural barrier to the expansion of PrEP services was the fragmentation of healthcare records and services. These aspects manifested in worries participants had about undergoing tests which would not be joined up with other health data and the need to repeat tests. Participants also expressed concern about how there is not a standard process which underpins the journey from consultation to prescription collection. The latter aspect was highlighted as being particularly problematic for people with multiple health conditions.

‘If I'm going to have a load of tests done at one point, I want to know that they are being integrated (into healthcare records) eventually, and that I'm not going to have records at one pharmacy where I'd had a sexual health check, or access PrEP or a vaccine or something, and it's not being sent over to my main sexual health clinic…I don't want to have to be chasing individual pharmacies for results. Or like when I was going away, for a vaccine, not having someone that has my full medical history, and then having to try to remember what I have had done.’

PID 19, Group 1, Cardiff, Aged 25–30, White, Man, Gay or bisexual

‘I've got ADHD…so I have three different prescribers, same health authority, three different places, three different regimes of how to collect and get the prescriptions…none of them are joined up…I feel like I am three patients. I feel like a patient with my GP, I feel like a patient with the sexual health clinic, I'm a patient with my mental health team…’

PID 5, Group 1, outside Cardiff, Aged 41+, White, Man, Gay or bisexual

## 4 Discussion

### 4.1 Summary of Main Findings

**Figure 2:**
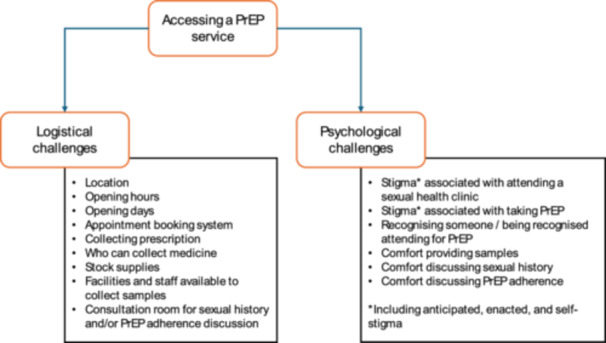
Decomposition of ‘access’ to PrEP services.

This is the first study from Wales, and one of the first across the United Kingdom, to explore prospective user views of a PrEP service in community pharmacies before implementation. We found broad acceptance of the idea of a PrEP service being delivered in community pharmacies. The key appealing features of such a service would be improvements in access—both in terms of the varied operating hours of community‐based services (e.g., evenings and weekends) and the broad use of such services reducing the stigma attached to attending. These were important barriers to access highlighted when descriptions were given of PrEP access via sexual health clinics. Thus, ‘access’ to PrEP services, regardless of setting, comprised two aspects—logistical challenges of gaining access to a health service (e.g., location and opening hours) and the psychological challenges associated with accessing a service (e.g., varying components of stigma, including anticipated, enacted and self‐stigma) (see Figure 2). Important positive aspects were noted regarding PrEP services delivered via sexual health clinics. These included well‐trained staff experienced in having sexual health discussions, the holistic nature of the sexual health service (e.g., PrEP prescription, STI testing, blood tests, other STI prevention or treatment services) and a sense of comparative anonymity when attending a sexual health clinic due to its location (e.g., out of a person's local area). Participants expressed uncertainty around the capacity and capability of community pharmacies and pharmacists to conduct certain aspects of healthcare (e.g., blood sampling) necessary for a full PrEP service and wondered whether the convenience of a local PrEP service may be offset by the entirety of their sexual healthcare (and accompanying data) becoming fragmented. Therefore, while considered acceptable, there were several uncertainties and concerns expressed by participants which may need addressing before the successful rollout of any service.

### 4.2 Strengths and Limitations

Globally, there are ambitions to widen access to PrEP to increase uptake and reach, particularly in traditionally underserved groups [25]. Differences in policy decisions regarding ways in which PrEP has been rolled out (e.g., universally vs. through a programme of research) may have led to differences in access. Our community‐based sampling approach has allowed us to include a diverse sample, incorporating views and experiences from individuals across Wales, different age groups, gender identities, sexualities and ethnic groups. We incorporated established frameworks to both include views of individuals at different stages of PrEP need and provide a structure around the study of access to healthcare. These informed the interviews and analysis and supported the justification for including individuals who have a self‐identified PrEP need but have not yet accessed it. Views from these individuals were important to incorporate if this study is to inform service provision for new as well as established PrEP users.

An important drawback to our community‐based sampling strategy was that it attracted so‐called ‘imposter participants’. The phenomenon of imposter participants has received increasing attention in qualitative research recently and seems to have coincided with an increase in the normalisation of remote interviews.

### 4.3 Comparisons to Existing Literature

Our findings are largely in line with a recent publication of community members' views of the barriers and facilitators of community pharmacy PrEP delivery conducted in England, the United Kingdom, and a wider scoping review of barriers and facilitators of community pharmacy PrEP delivery [26, 27]. Key differences include the choice of models/framework used (COM‐B [28] vs. PrEP need framework and Levesque framework for healthcare access [17]), geographical variation (e.g., healthcare is devolved in Wales) and policy contexts within which studies were conducted both in terms of how PrEP was rolled out and how community pharmacies operate. For example, PrEP was initially made available in England as part of a research study with a cap on the numbers of those who could access it, whereas, in Wales, it was available without any cap on numbers to those who met initial clinical indications. Additionally, community pharmacies in Wales have offered clinical services for more than a decade, whereas the structure underpinning this across is more of a recent initiative in England [14, 29]. Additional differences are present in the respective topic guides, with the work by Harrison et al. [26] including more direct questions and fewer questions about interviewees' experiences of PrEP access via sexual health clinics and broader experiences of healthcare access via community pharmacy. Importantly, in our findings, we found that the acceptability of a community pharmacy PrEP service is likely to be differential and depend on both an individual's wider healthcare needs and psychological concerns regarding sexual healthcare access. Thus, these studies are likely to be complementary and give some understanding of the role of context (particularly around PrEP rollout and community pharmacy set‐up) on influencing factors around any new PrEP services.

### 4.4 Implications

There is a need for an ongoing understanding of the acceptability of the expansion of healthcare services to community pharmacies. As new services are rolled out, they need to be subject to ongoing training, monitoring and evaluation (including economic evaluation), which also captures the experiences of those accessing services. Furthermore, the nature of community pharmacies as both a healthcare setting and a business may have implications with regard to client/customer expectations and trust. As the focus of this paper is on the prospective users' influencing factors, we did not consider the structural context of service delivery such as commissioning and primary care drug delivery costs. Additionally, the deployment of remote community‐based qualitative research requires safeguards against imposter participants. This is particularly problematic for topics such as sexual health, as participants particularly value anonymity and may be more guarded (or unwilling to participate) if verification of inclusion criteria is seen to be overly intrusive. Care is required for the use of data collection methods which do not limit the inclusion of underserved populations. For practice, any rollout of a PrEP service via a community pharmacy, or any other setting outside of a specialist sexual healthcare service, must be twinned with a communication strategy which informs people what PrEP is, where it can be accessed and what to expect as part of any access (including how data are communicated to relevant parties). Furthermore, staff delivering the PrEP service must be trained to engage in sexual health discussions in a non‐judgemental manner, displaying cultural competency, sensitivity and inclusivity across a range of gender identities and ethnic groups. A rollout that does not include the type of communication and training outlined here will have limited impact and likely only lead to a partial displacement of PrEP users from current services.

## 5 Conclusion

In our study, we found that PrEP access via community pharmacy is likely to be an acceptable option for potential users. It would reduce current barriers for some—particularly those who find it difficult to access sexual health clinics due to their location, operating hours or stigma. There are uncertainties surrounding what a PrEP service should and could look like in community pharmacies, and any successful rollout should be accompanied by clear communication to maximise its impact.

## Author Contributions

**David Gillespie:** conceptualization, investigation, funding acquisition, writing – original draft, methodology, writing – review and editing, formal analysis, project administration, data curation. **Adam D. N. Williams:** conceptualization, investigation, writing – review and editing, formal analysis, data curation, methodology, visualization. **Richard Ma:** funding acquisition, writing – review and editing, formal analysis, data curation, conceptualization. **Zoe Couzens:** conceptualization, funding acquisition, writing – review and editing. **Kerenza Hood:** conceptualization, funding acquisition, writing – review and editing. **Dyfrig A. Hughes:** conceptualization, funding acquisition, writing – review and editing. **Efi Mantzourani:** conceptualization, writing – review and editing, methodology, investigation. **Eleanor Cochrane:** writing – review and editing, methodology. **Fiona Wood:** conceptualization, investigation, funding acquisition, writing – review and editing, methodology, formal analysis, data curation.

## Ethics Statement

The study was reviewed and approved by the Cardiff University School of Medicine Research Ethics Committee (Reference Number: 23/91).

## Conflicts of Interest

D.G. reports receiving funding from Health and Care Research Wales during the conduct of this study. K.H. also reports a leadership role for Cardiff University on the Fast Track Cardiff & Vale leadership group. A.W. reports a role on the Fast Track Cardiff & Vale steering group, as does D.G., who also reports a role on the Fast Track Cymru Advisory Council along with Z.C. These groups are local branches of the Fast Track Cities initiative aiming at eradicating HIV by 2030. D.G. and Z.C. are also members of the Wales HIV Action Plan Implementation group.

## Data Availability

Data (thematic coding matrices for all developed themes) are included as an appendix attached to this publication. Due to privacy/ethical restrictions, other data (e.g., full transcripts) are not available.

## Appendix 1

**Topic guide**

*[Probe based on PrEP need framework (PrEPster, 2023). For example, probe topics such as stigma, lack of control, acceptability of available/potential PrEP services, access issues in terms of opening times and geography, denial of PrEP, or access issues regarding restarting PrEP. Questions will be framed around the Healthcare Access Framework (Levesque* et al., *2013)]*

**Opening**

My name is <Interviewer> and I am <DO‐PrEP role> for the DO‐PrEP project.

I would like to ask you some questions about your experiences of accessing PrEP.

I hope this information will help better understand the experiences people have of accessing PrEP in Wales, and the acceptability of alternative PrEP services which may be available in Wales in the future.

The interview should take about 30–60 min.

Are you able to respond to some questions at this time?

**Consent**

Before I start, please can you confirm the following:

<Consent clauses>

**Body**

I'd like to start by asking about your previous experiences of accessing PrEP.

**Topic 1:** familiarity with PrEP and prior experiences accessing PrEP (PrEP access history)

You've previously told me that <prior experience of taking PrEP>, could you tell me a bit more about this (e.g., how you have found accessing PrEP through sexual health clinics)?

***Suggested specific sub‐questions:***

How/did you hear that PrEP was available in sexual health clinics?

How do you feel about having to access PrEP only through sexual health clinics in the NHS in Wales?

Are there any (any other, if already covered in part) positives about accessing PrEP through a sexual health clinic for you?

Are there any (any other, if already covered in part) negatives about accessing PrEP through a sexual health clinic for you?

How important is it to you that you don't have to pay for your PrEP prescription or appointments about PrEP?

Now I'd like to move on and ask you questions about other settings where PrEP may be made available in the future. We're going to start with community pharmacies.

**Topic 2: PrEP access in community pharmacies**

Can you tell me what you know about community pharmacies and the services they provide?

Specific probes around health literacy, beliefs, trust, expectations, personnel and confidentiality.

Have you visited a community pharmacy before?

If so, what sorts of things have you been to a community pharmacy for?

What do you find convenient about community pharmacies?

*Specific probes around living environment, transport/mobility, social support, also cover hours of opening, personnel, confidentiality*.

What do you find inconvenient about community pharmacies?

*Specific probes around living environment, transport/mobility, social support, also cover hours of opening, personnel, confidentiality*.

What are your thoughts on PrEP services being delivered through community pharmacies?

*Specific probes around personal and social values, culture, stigma, autonomy, personnel and confidentiality*.

Thinking about the answers you have given about community pharmacies generally, are there any further points you'd like to make about the convenience or inconvenience of PrEP services being available in this setting?

I'd like to now ask about your thoughts on the delivery of specific aspects of PrEP services in community pharmacies. What are your thoughts on:

PrEP collection

STI screening

HIV screening

Renal monitoring

Sexual health history

Risk counselling

Adherence counselling

I'd now like to move on and ask you questions about PrEP service delivery in other settings.

**Topic 3: PrEP access in other community settings**

What other community settings would you like to see offering PrEP services?

For each setting mentioned:

Why this setting in particular?

What PrEP services do you think this setting should deliver?

What might be the advantages of PrEP services being available in this setting?

Do you think that offering PrEP in this setting will be good value for money for the NHS in Wales?

What might be the disadvantages of PrEP services being available in this setting?

*Specific probes on domains mentioned above (health literacy, beliefs, trust, expectations, living environment, transport/mobility, social support, also cover hours of opening, personal and social values, culture, stigma, autonomy)*

**Close**

Is there anything else you would like to talk about regarding PrEP access in Wales?

*Stop recording*

Thank you for the time you have given today. <Confirm contact details for incentive>.

## Appendix 2

Thematic matrices

Table A2

**Table A2:** Experience in accessing PrEP through sexual health clinics.

Initial coding	Sub‐theme	Example of a direct quote
Pathways to accessing PrEP via sexual health clinic	Process of getting PrEP through a sexual health clinic	‘So, first, I'm a sex worker. So, I need to protect myself, because I do have a lot of clients with several requests. And in my line of work, I have a very high risk of contracting the virus. So, I have to take tests to protect myself, and one of these tests was getting PrEP. I have unprotected sex with my clients and this was readily available to me when I do have sex, through my local community Sexual Health Clinic. This was available on trial.’ ***PID 3**, Group 2, outside Cardiff, Aged 25–30, non‐White, Man, Straight* ‘I heard from friends initially so when I went for a check at the (general) health centre, so I asked about the PrEP medication. They told me all about it and how I can get it and the prescription for it.’ ***PID 16**, Group 3, Cardiff, Aged 25–30, non‐White, Woman, Straight*
Ease of access to PrEP via sexual health clinics		‘I remember the hardest thing for me was being able to attend the clinic, because I work Monday to Friday basically nine until five and I think the clinic is open nine until three and it's not open five days a week, or it wasn't at the time. The only way for me to actually be able to attend in‐person was to take time off work, which felt to me like something that wasn't a very logical thing. Many of us work those hours and struggle to get to a clinic because of that.’ ***PID 1**, Group 2, Cardiff, Aged 25–30, White, Man, Gay or bisexual* ‘I had to go to one hospital to have the check‐up and to have the request for it, and all of those things … they gave me a prescription, but then I had to go to another hospital (to) their pharmacy to collect it … they were giving three months' supply, so … every three months I have to go through the same process.’ ***PID 5**, Group 1, outside Cardiff, Aged 41*+, *White, Man, Gay or bisexual* ‘I think trying to get (access to) the service … that's from finding about it and researching and booking the call and being able to find appointments, the process was not stressful so that's kind of a positive.’ ***PID 14**, Group 1, outside Cardiff, Aged 31–40, non‐White, Man, Gay or bisexual*
Awareness of inconsistencies across health boards		‘… And I think the inconsistency whether you're in (Health Board X), whether you're in (Health Board Y), whether you're (in) (Health Board Z), I think it shouldn't be … you know, whether you're in McDonalds in (Area X) or in McDonalds in whatever, they might have different seating … if it was a franchise. But you still get a Big Mac, it's still the same (service), and I think the problem is that whilst you can appreciate that there might be different franchise holders, it does feel like it's (a) different menu, it does feel like it's different things, and that's difficult I think.’ ***PID 5**, Group 1, outside Cardiff, Aged 41*+, *White, Man, Gay or bisexual* ‘Well in (area) so say if you lived in (area) you've got to drive all the way to (hospital), have the consultation because that's where the GUM clinic is, and then they will write a prescription and take it to, erm, so you can take it to any hospital pharmacy, but if you were living in (area), you'd bring it to the (different hospital), whereas in (different area) they've got the pre‐labelled, erm, boxes, so you get it, er, straight away, erm, and I've raised this within (health board) multiple times because I find it such a barrier for patients here.’ ***PID 8***, *Group 2, Cardiff, Aged 31–40, White, Man, Gay or bisexual*
Privacy issues and concerns around access to PrEP via sexual health clinics	Anticipated and enacted stigma and discrimination around PrEP access via sexual health clinics	‘*I don't like to go there and get lots of questioning and all that, so, it's one of the barriers, that's kind of, hindering me.’ **PID 4,** Group 2, Cardiff, Aged 25–30, non‐White, Woman, Straight* ‘It's not a problem for me, but I know that there is still a stigma attached to sexual health clinics. So, I don't know whether having to get PrEP through a sexual health clinic would put people off. Not wanting to just … (share) their sexual orientation, or the fact that they are engaging in sexual experiences that might require PrEP.’ ***PID 19**, Group 1, Cardiff, Aged 25–30, White, Man, Gay or bisexual*
Attitudes of staff		‘Yeah, the good aspect is that for me the person I got it (PrEP service) from, he gave me the original or a good prescription. Positively, he's been relating with me in a good manner … you know, I'm a person, I need PrEP.’ ***PID 10**, Group 1, Cardiff, Aged 31‐40, non‐White, Man, Straight* ‘I'm not judging the clinic (in relation to perceived racial profiling by clinic staff), I might actually be judging the staff that's actually working there because we (PrEP service users) come there with a different mindset and they (staff) have their own mindset, different from us, so it might not be about the job, it might be about how, what they believe, how they see people and how they understand certain situations.’ ***PID 11,** Group 1, outside Cardiff, Aged 31–40, non‐White, Man, Straight*
Assessment of risk and ‘PrEP eligibility’		‘I didn't enjoy the whole communication in seeking out (information) because they made me really focus on so many personal things such as my sexuality, and I think those questions going around in healthcare might really be best to me … I think the whole process of the referral from a sexual health clinic shouldn't be necessary because they (clinic staff) see it as an opportunity to ask a whole lot of questions that I don't think is necessary.’ ***PID 2,** Group 2, Cardiff, Aged 25–30, non‐White, Man, Straight*
Expectations of service		‘Well, it's (having access to PrEP in a sexual health clinic) positive because… I would assume the most common way for the virus to be transmitted is through sexual means. So, it absolutely makes sense that the PrEP medication would be available at the Sexual Health Clinic.’ ***PID 3,** Group 2, outside Cardiff, Aged 25–30, non‐White, Man, Straight* ‘I trust the NHS in Wales and I trust that I'm being a customer, a user of (clinic). The personal experience, the medical staff, and the administrative staff as well, has been excellent.’ ***PID 6**, Group 1, Cardiff, Aged 41*+, *White, Man, Gay or bisexual*
**Prospect of accessing PrEP through community pharmacies**		
Awareness of community pharmacies and their services	Awareness of community pharmacies as a healthcare setting	‘…you know (the type of services provided by community pharmacies), medication dispensing, and (the) primary function of these pharmacies (are to dispense) prescriptions and (provide) immunisations and also most of these pharmacies hold screening and component services.’ ***PID 12,** Group 2, outside Cardiff, Aged 31–40, Other demographic details missing* ‘I've noticed recently that services that I would normally have gone to my GP for have been deferred to my pharmacy…I would think it's (community pharmacy) where I go to get my prescription, it's where I could go for some advice if I don't need to go to A&E and I don't feel like I desperately need to see a doctor, but I probably wouldn't go there for much else.’ ***PID 20,** Group 3, Cardiff, Aged 31–40, White, Man, Gay or bisexual*
Business of community pharmacies		‘I do have a distinction between community pharmacy and the NHS…The NHS is a public service. Whereas the community pharmacy is a private sector…If I wanted a quality service from a community pharmacy, I would go to (major high‐street community pharmacy). I trust (major high‐street community pharmacy) more than the community pharmacy I use… If I was ill, or if I had a complaint, or if there was something I felt wrong, I wouldn't go to community pharmacy, I would (web search) NHS, or ring 111…Community pharmacy to me…it's a shop that sells things.’ ***PID 6,** Group 1, Cardiff, Aged 41*+, *White, Man, Gay or bisexual*
Access to community pharmacies	Experience of service delivery through community pharmacies	‘Obviously pharmacies are stretched as well at the moment and sometimes they can be really busy but the advice that you get from people is really helpful and it's a lot easier than getting a GP appointment or finding the information you need online, lots of the time.’ ***PID 7,** Group 1, Cardiff, Aged 25–30, White, Man, Gay or bisexual* ‘The one (convenient point about community pharmacies) closest to me is that it's open on a Saturday, so if I can't get there Monday to Friday, I can at the weekends.’ ***PID 1**, Group 2, Cardiff, Aged 25–30, White, Man, Gay or bisexual*
Stock issues		‘Sometimes they (community pharmacies) run out of a particular drug so then you come and it's not available. This was I think last month. I came to get a particular medication, and they didn't have it. They are usually out of medication really quick.’ ***PID 14,** Group 1, outside Cardiff, Aged 31–40, non‐White, Man, Gay or bisexual*
Trustworthiness and familiarity of community pharmacy staff and setting		‘I think a pharmacy has less people and … being somewhere you go often for, maybe for a different purpose, you might feel much more comfortable in that kind of place… I think the familiarity is there and it makes it easier to feel much more comfortable.’ ***PID 11,** Group 1, outside Cardiff, Aged 31–40, non‐White, Man, Straight*
Training of community pharmacy staff	Delivery of a ‘PrEP service’ through community pharmacies	‘I would suggest the pharmacies are more sensitised about PrEP…they should be given more knowledge, more awareness about it, and more education about PrEP and its benefits. And there should be thought how to create a welcoming environment, and a less judgemental environment for people that want to access PrEP.’ ***PID 17,** Group 3, Cardiff, Aged 25–30, non‐White, Man, Straight*
Confidentiality and privacy concerns around community pharmacies		‘I think the service you get at a community pharmacy and whatever the medication is you're picking it up, it's usually confidential. It obviously depends on if there's questions to ask as well. They've always got a booth if they need to ask questions in there. From a stigma point of view, which there is around PrEP and HIV and things still, but maybe that's more of a generational thing as well, where I feel like my generation are quite open about those kinds of things and I see that as a positive accessing PrEP, and something I talk openly to people about.’ ***PID 7,** Group 1, Cardiff, Aged 25–30, White, Man, Gay or bisexual* ‘Usually when I get into this pharmacy, the conversations are usually never lengthy, they are almost straight to the point. I come in and get what I am looking for, fill in a prescription and then … we usually don't converse or talk about anything …it's usually very, very straight forward. So, it doesn't get to the point where you get asked unnecessary questions or you get dragged into a conversation that would have you start thinking about your privacy. … Because I would say they are very close to me, I wouldn't feel so comfortable having an STI screening in a community pharmacy. Mainly because of the proximity. This is how I feel about it, I wouldn't feel so comfortable, so I'd prefer to go a 30 minutes' drive to get my STI screening than walk into a pharmacy.’ ***PID 14,** Group 1, outside Cardiff, Aged 31–40, non‐White, Man, Gay or bisexual*
Perceived pharmacist discomfort/judgement		‘I worry that there might be some pharmacists that wouldn't do the training because of the stigma associated with HIV…there are some pharmacists that refuse to do the morning after pill because if they're Catholic or Muslim it goes against religion…And I worry that would happen with PrEP as well, because it could be seen as men having sex with men…’ ***PID 8,** Group 2, Cardiff, Aged 31–40, White, Man, Gay or bisexual* ‘I wouldn't have a problem with having that conversation (about PrEP) with somebody there (a community pharmacy) as long as I had faith that they (were) trustworthy and non‐judgemental and gave me a private space to have that conversation openly.’ ***PID 20,** Group 3, Cardiff, Aged 31–40, White, Man, Gay or bisexual*
Facilities for consultations and testing		‘I would want to know how it would work, because the first time I went on PrEP, I had the blood test taken and I don't feel that can be done at a pharmacy. So, at some point you will need to be in either a sexual health clinic, or a GPs surgery, where blood work can be done. Because that seems to be part of accessing it…but if once that has been done…I almost want a certificate that I can take in the pharmacy, where the GP goes or the sexual clinic goes you are fine for your kidney works, you are now able to access PrEP. … …it was useful for me to go to the sexual health clinic because I was able to knock out everything at once. I had all the tests completed. I could see that being an issue at pharmacy, where you couldn't get everything integrated….as well I was able to pick up contraceptives at the (sexual health) clinic too after I'd had all the tests…It'd be like here's your PrEP, here's your condoms, have a great time.’ ***PID 19,** Group 1, Cardiff, Aged 25–30, White, Man, Gay or bisexual*
PrEP collection		‘…picking up a prescription is different from going up to a public counter and going “can I have some PrEP please?” … I think it (people's comfort around “accessing PrEP via community pharmacies”) depends on whether they're going to the pharmacist for advice, whether they're having some form of consultation, or some form of patient direction. I think that's different to going to the pharmacy directly, to pick up a prescription, that's understood, known, dispensed, and you're just picking up a package, because you could be picking up anything then, it doesn't really matter. If I was to go to the pharmacy and try to have a conversation I might be having at the sexual health clinic in the pharmacy, I think that might be challenging.’ ***PID 5,** Group 1, outside Cardiff, Aged 41*+, *White, Man, Gay or bisexual* ‘If it (PrEP) were to be delivered from pharmacies…people would be able to access it easily.’ ***PID 16,** Group 3, Cardiff, Aged 25–30, non‐White, Woman, Straight*
STI testing and other testing (e.g., liver and kidney function testing)		‘I can't think I'd have a problem with that (STI testing services) being in a pharmacy setting. It comes back to the same thing if you trust the practitioner who is helping you and you need the service, just do it…It would be a little uncomfortable, a little awkward sometimes but you just get on with it for the sake of your health right.’ ***PID 20,** Group 3, Cardiff, Aged 31–40, White, Man, Gay or bisexual*
Counselling/advice around sexual health/medication adherence		‘…it depends on how my conversation with the person is going to be, and client confidentiality. So that would be a major factor around how I would approach sharing my sexual history with a community pharmacy. So, it depends, it really, really depends. If I have some form of assurance on how my conversation, my data is going to be handled then that would help on how I approach and share information with the community pharmacy…. these types of conversations (adherence counselling) are not so intimate; therefore, I would be okay to have these kinds of conversations because they are conversations about how to use the medications properly and so it's not so private.’ ***PID 14,** Group 1, outside Cardiff, Aged 31–40, non‐White, Man, Gay or bisexual*
**Prospect of accessing PrEP through other community settings**		
GP practices	PrEP delivered via general practice	‘…unless there's something I'm unaware of, I could get this, just a prescription from a, from a GP’ ***PID 6,** Group 1, Cardiff, Aged 41*+, *White, Man, Gay or bisexual* ‘…it feels a bit backwards, the fact you can get the contraceptive pill through GP, whereas you can't get PrEP through GP, just to me it doesn't make sense. Blood test wise you, you can get that easily through the GP surgery…so it (PrEP) could easily be retrieved from the community pharmacy’ ***PID 8,** Group 2, Cardiff, Aged 31–40, White, Man, Gay or bisexual*
Trans clinics	Other specialist health services	‘…maybe linking in with trans healthcare, those settings could also be a place where they um, they have PrEP available…for a lot of trans‐people, talking to a clinician that you know is well‐trained in kind of matters of trans‐identity and experience is possibly the only way that some trans‐people might actually consider accessing PrEP…any other less specialist services, whether it is your GP or your sexual health clinic, or your pharmacy, it does always feel like more of a risk of like putting yourself out there in a position where you might have to educate the people that are supposed to be more educated than you about that kind of medical thing that you're seeking support with.’ ***PID 1,** Group 2, Cardiff, Aged 25–30, White, Man, Gay or bisexual*
Mental health services		‘…because these (mental health) services are going to be handled by (a) professional, maybe a therapist who is a mental (health) therapist, counselling people of the different mental illness. Sometimes someone that is suffering from a sexual illness can have mental sickness or mental issues that affect his or her thinking……apart from the PrEP services also they can give an avenue for counselling, consultation and, you know, examination.’ ***PID 10,** Group 1, Cardiff, Aged 31–40, non‐White, Man, Straight*
Hubs/libraries	Community‐based organisations	‘I know across (the local area) there's more plans for wellbeing hubs and health centres and things over the next ten years so they'd seem like a logical place to offer some of those things (PrEP services)’ ***PID 7,** Currently accessing PrEP through sexual health clinic in Wales, Cardiff, Aged 25‐30, White, Man, Gay or bisexual* ‘I don't know if there would ever be the demand for it but potentially you could have it in local hub settings.’ ***PID 19,** Group 1, Cardiff, Aged 25–30, White, Man, Gay or bisexual*
Supermarkets		‘Supermarkets…I mean there are many precedents, if you think about erectile dysfunction and you can now buy Viagra or the equivalent off the shelf. And, I believe Viagra can have some adverse side effects, particularly those that have blood pressure issues, or those that combine use of Viagra with recreational drugs…But I assume that somehow they've overcome that barrier by actually selling them on supermarket shelves…And, so, therefore, if, PrEP is safe, which I think it is, and if PrEP can result in improvement or safeguard the individual health and general collective public health, why wouldn't you make it as open and accessible to everyone?’ ***PID 6,** Group 1, Cardiff, Aged 41*+, *White, Man, Gay or bisexual*
Mobile/travel clinics	Digital services/decentralised PrEP	‘You know, banks have closed down and in my small village, a bank van comes out once a week and you know what time it gets there, it stops in the layby, and for two hours it's there, and you could quite easily have a sexual health van.’ ***PID 6,** Group 1, Cardiff, Aged 41*+, *White, Man, Gay or bisexual*
Digital services and home‐based PrEP		‘You (could) actually do it (access PrEP) in the convenience of your home and really order the drug to your home address would really be good…So more like test kits being posted to your home address, conveniently would help. It would definitely ensure that you're always on track about your PrEP medication. It will increase aspects of healthcare and eliminate waste, waiting times…the advantages outweigh the disadvantages.’ ***PID 2,** Group 2, Cardiff, Aged 25–30, non‐White, Man, Straight*
**Integrated health services and record linkage**		
Handling other health conditions	Fragmented healthcare and records	‘I've got ADHD…so I have three different prescribers, same health authority, three different prescribers, three different places, three different regimes of how to collect and get the prescriptions…none of them are joined up … I feel like I am five patients. I feel like a patient with my GP, I feel like a patient with the sexual health clinic, I'm a patient with my mental health team…’ ***PID 5,** Group 1, outside Cardiff, Aged 41*+, *White, Man, Gay or bisexual*
Joined up health records		‘I would be happy for it (sexual history taking and risk counselling in community pharmacy), but I think it has to with the point we touched on earlier about record integration…If I'm going to have a load of tests done at one point, I want to know that they are being integrated eventually, and that I'm not going to have records at one pharmacy where I'd had a sexual health check, or access PrEP or a vaccine or something, and it's not being sent over to my main sexual health clinic, if that makes sense…I don't want to have to be chasing individual pharmacies for results. Or like when I was going away, for a vaccine, not having someone that have my full medical history, and then having to try to remember what I have actually had done.’ ***PID 19,** Group 1, Cardiff, Aged 25–30, White, Man, Gay or bisexual*
